# Rapid Genotyping of *FecB* Mutation in Sheep Using CRISPR-Cas12a Integrated with DNA Nanotree Biosensing Platform

**DOI:** 10.3390/biom15030315

**Published:** 2025-02-20

**Authors:** Dongxia Pan, Mukaddas Mijit, Hui Wang, Chaoqun Sun, Bantan Pingcuo, Zhixue Yu, Benhai Xiong, Xiangfang Tang

**Affiliations:** 1 State Key Laboratory of Animal Nutrition and Feeding, Institute of Animal Science of CAAS, Beijing 100193, China; pandongxia1117@163.com (D.P.); muukaddas@163.com (M.M.); wanghui10@caas.cn (H.W.); 82101235459@caas.cn (C.S.); 82101205310@caas.cn (Z.Y.); 2 Institute of Animal Husbandry and Veterinary, Tibet Autonomous Regional Academy of Agricultural Sciences, Lhasa 850004, China; 82101235461@caas.cn

**Keywords:** CRISPR-Cas12a, amplification retarded mutation system, *FecB*, Fe_3_O_4_@Au-Linker-Cy5-Nanotree, rapid detection

## Abstract

The A-to-G mutation (*FecB*) in the *BMPR1B* gene is strongly linked to fertility in sheep, significantly increasing ovulation rates and litter sizes compared to wild-type populations. The rapid and reliable screening of the *FecB* gene is therefore critical for advancing sheep breeding programs. This study aimed to develop a fast and accurate method for detecting the *FecB* mutation and genotyping the gene to enhance sheep reproduction and productivity. To achieve this, we integrated the CRISPR-Cas12a system with an optimized amplification refractory mutation system (ARMS). A similar DNA origami technique-based fluorescence reporter nanotree structure was synthesized using gold nanomagnetic beads as carriers to amplify the fluorescence signal further. The resulting biosensing platform, termed CRISPR-ARMS, demonstrated excellent sensitivity for detecting *FecB* mutations, with a detection limit as low as 0.02 pmol. Therefore, this innovative approach shows great promise for single-base mutation detection and represents a pioneering tool for high-yield genetic screening.

## 1. Introduction

Multiple births in mutton sheep are a key economic trait in the breeding industry, contributing 74% to 96% of the industry’s economic benefit. As a result, lambing traits have garnered significant attention as a critical factor in mutton sheep breeding [1]. Numerous studies have shown that FecB mutation sites are crucial in enhancing ovulation rates and lambing numbers in meat sheep. In Hu sheep and small-tailed Han sheep, *FecB* mutations primarily increase ovulation rates and lamb numbers. Ewes with GG and AG genotypes produce 1.40 and 1.11 more lambs, respectively [2]. Sheep breeds carrying the *FecB* gene exhibit high fertility, providing a direct marker for detecting *FecB* DNA mutations. Ovulation rate and lamb number are the main factors influencing sheep reproductive capacity, and traditional breeding methods based on phenotypic data are slow to improve these traits. Therefore, marker-assisted selection and molecular genetics are crucial for advancing reproductive efficiency [3]. To date, methods such as PCR-Sanger sequencing [4], TaqMan probe [5], restriction fragment length polymorphism (RFLP), and single-strand conformation polymorphism (SSCP) [6] have been developed to detect FecB mutations. However, these methods require extensive manual labor or are unsuitable for high-throughput detection, and they demand skilled operators. Therefore, a faster, cost-effective, and user-friendly detection method is still needed.

The CRISPR-Cas system has significant potential for detecting nucleic acid with single or multiple base mutations. In a suitable buffer, Cas protein and single-guide RNA (sgRNA) spontaneously assemble into Cas ribonucleoproteins (CasRNP). Based on the principle of complementary base pairing, sgRNA binds to the target double-stranded DNA (dsDNA). When a protospacer adjacent motif (PAM) is present, the Cas endonuclease is activated, cleaving the dsDNA and creating dsDNA breaks [7]. Among the Cas variants, Cas12a recognizes T-rich PAM sequences (TTTV) and catalyzes the maturation of self-guiding RNA (crRNA) [8]. This process results in the cleavage of dsDNA distal to the PAM site, producing 5′ and 3′ sticky ends [9]. Additionally, the Cas12a/crRNA/target DNA ternary complex exhibits non-specific single-strand DNA cleavage activity upon formation [10]. Leveraging this property, Wang Mengyu et al. [11] developed Cas12aFVD (Rapid Visual Detection), a portable and efficient biosensing platform based on the non-specific single-strand DNA-cutting activity of CRISPR-Cas12a to detect single base mutations in gene-edited rice. This system successfully enabled single nucleotide polymorphism (SNP) typing in rice. Similarly, Fu Jinyu et al. [12] reported a fluorescence biosensor based on the CRISPR-Cas12a system. By integrating target-specific cleavage with a DNA toehold-mediated strand displacement (TMSD) reaction, they demonstrated the system’s single nucleotide specificity using the epidermal growth factor receptor (EGFR) gene from non-small cell lung cancer (NSCLC) as a target. Differences in fluorescence signals between wild-type and mutant targets validated CRISPR-TMSD’s single nucleotide specificity.

With advancements in nanotechnology and nanoscience, various nanomaterials have been developed to enhance the sensing performance of electrochemical biosensors [13] or serve as signal amplification tools [14]. Currently, nanomaterials such as gold nanoparticles, silica, iron oxide, and magnetic particles exhibit exceptionally high detection sensitivity, primarily for detecting proteins and nucleic acids [15]. Among these, gold nanomagnetic beads (Fe_3_O_4_@Au), a type of stable metal nanomagnetic bead, have garnered significant attention as nanocarriers due to their favorable properties, including excellent biocompatibility, ease of preparation and modification, and large surface area [16]. The size of these magnetic beads is critical, as it determines the surface area available for fluorescent probe binding. A larger surface area enables the attachment of more fluorescent probes, thereby amplifying the signal [17]. Additionally, organic thiol compounds and thiolated oligonucleotides react with the surface of gold nanomagnetic beads to form Au-S covalent bonds, further enhancing the biosensor’s sensitivity and selectivity [18].

DNA origami technology is based on the nanoscale folding of long, single-stranded DNA skeleton chains using hundreds of complementary anchoring strands [19]. This method offers several advantages, including ease of operation, high assembly efficiency, rapid reaction rates, high experimental success rates, simple implementation processes, and nanoscale addressability [20,21,22]. These features facilitate the construction of complex yet controllable nanostructures with stable, precise, and accurate architectures [23,24,25]. Recent advancements in DNA manipulation by researchers have driven the exploration of DNA origami from one-dimensional to two-dimensional structures, and further into three-dimensional configurations. These developments have expanded its applications to diverse fields such as biological computing, biosensors, pharmaceutical carriers, and information security [26,27]. Despite its versatility, DNA origami technology typically relies on single DNA strands with thousands of bases as backbones, which are costly to synthesize. To address this, we employ a modified DNA origami approach, utilizing single or multi-stranded DNA sequences of varying lengths. By leveraging hybridization and the Watson–Crick base pairing principle, we construct 1D to 3D nanoscale devices and structures capable of fluorescence signal amplification. Compared to traditional DNA origami methods, this approach reduces synthesis and assembly costs while minimizing reliance on long DNA sequences.

**Scheme 1:**
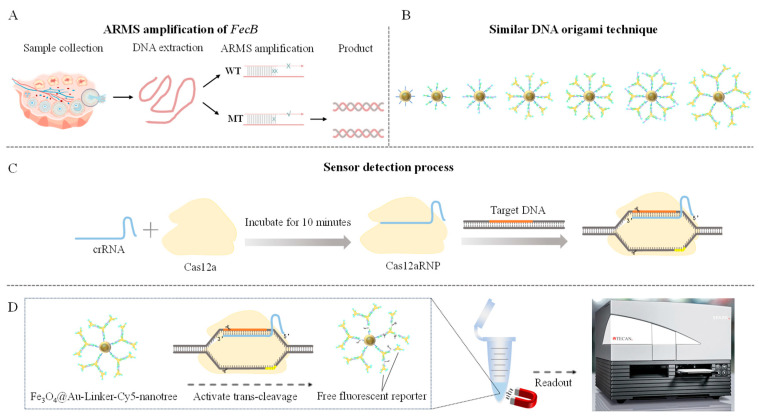
Schematic diagram of CRISPR-ARMS assay to distinguish FecB alleles. (**A**) ARMS amplification of *FecB* allele. (**B**) Similar DNA origami techniques. (**C**) Construction process of Cas12aRNP. (**D**) CRISPR-ARMS system test procedure.

ARMS technology relies on the allele-specific extension response. The extension reaction occurs only when the 3′ terminal base of an allele-specific primer complements the base at the mutation site. This method is characterized by simplicity, high sensitivity, and low cost [28]. In this study, we developed a rapid, highly accurate biosensing platform for genotyping *FecB* mutated genes. In the CRISPR-ARMS system targeting the *FecB* allele, the last base at the 3′ end of the upstream primer is G, with an additional mismatch introduced at the second or third base of the upstream 3′ end. Four pairs of ARMS-specific primers were designed to evaluate the sensitivity of the mutation site, and the optimal primers were selected for specific amplification of the *FecB* mutant gene. The precise binding of Cas12a/crRNA to the target DNA activates Cas12a’s trans-cleaving activity, enabling the cleavage of a fluorescent reporter gene. To amplify the fluorescence signal, a controllable fluorescent reporter gene nanotree structure was fabricated using gold nanomagnetic beads combined with DNA origami technology. The fluorescence signals were monitored using the Y040 multifunctional enzyme labeler (Thermo Fisher Scientific, Waltham, MA, USA). Both systems demonstrated excellent performance in detecting mutated *FecB* genes, establishing them as promising platforms for high-yield gene assays. The detailed process is shown in Scheme 1.

## 2. Materials and Methods

### 2.1. Materials and Reagents

Twenty-six ovarian genome samples (containing 18 validation samples) of wild-type and mutant *FecB* of small-tailed Han sheep were collected from the Hengshui test sheep farm in Hebei Province and cryopreserved. The animal study protocol was approved by the institutional review board of the Institute of Animal Science, Chinese Academy of Agricultural Sciences (protocol code IAS2024-180 and date 16 March 2024). Among them, BB represents mutant type, ++ represents wild-type, and B+ represents heterozygous type. Based on the Cas12a pronuclease spacer adjacent sequence (PAM) site, a crRNA was constructed to correspond to FecB mutant sequence and then synthesized by Integrated DNA Technologies (Coralville, IA, USA) and cryopreserved. Cas12a nuclease was obtained from Beijing Zeping Technology Company (Beijing, China). TCEP and ethanol were sourced from Shanghai Maclin Biochemical Technology Company (Shanghai, China), while Tris-HCl buffer with varying pH values was purchased from Leaf Biology (Shanghai, China). Sodium acetate was provided by Sigma-Aldrich (Beijing, China), and acetic acid (CH_3_COOH) was procured from Shanghai Aladdin Company (Shanghai, China). Reagents and kits included a magnetic bead-based animal genomic DNA extraction kit and an EZ-10 column DNA gel recovery kit, both purchased from Bioengineering (Shanghai, China) Company. Additional materials, including 2× SanTaq PCR Mix premix, DNA molecular weight standard marker (100–2000 bp), 50× TAE buffer, 10,000× 4S GelRed nucleic acid dye, 6× glycerol gel loading buffer VII, primers, and fluorescent reporter genes, were obtained from Shenggong Bioengineering (Shanghai, China) Company. The specific sequences are listed in Table 1. All experiments used ultra-pure water (Millipore, Burlington, MA, USA), and all chemical reagents were of analytical grade, requiring no further purification.

### 2.2. Genomic DNA Extraction and Genotype Detection

Eight genomic ovary samples representing *FecB* genotypes BB, ++, and B+ of small-tailed Han sheep were cryopreserved at −20 °C. The genomic DNA was extracted from the sheep ovarian tissue using a magnetic bead-based DNA extraction kit and stored at −20 °C. PCR amplification was performed in a 50 µL reaction system containing 5 µL of template DNA, 25 µL of high-fidelity PCR Mix premix, 2 µL of upstream and downstream primers, and 16 µL of ddH_2_O. The reaction conditions included an initial denaturation at 95 °C for 30 s, followed by 32 cycles of 95 °C for 15 s, 60 °C for 15 s, and 72 °C for 30 s, with a final extension at 72 °C for 5 min. Primer sequences for PCR are detailed in Table 1. PCR products were analyzed using 1% agarose gel electrophoresis, purified with the EZ-10 column DNA gel recovery kit, and subsequently sequenced.

### 2.3. Optimization and Detection of ARMS Primers

Distinguishing alleles in ARMS amplification using specific primers with a single nucleotide mutation at the 3′ end is generally challenging. To address this, ARMS-specific primers were designed separately for wild-type and mutant genes. The last base at the 3′ end of these primers was A or G, with additional mismatches introduced at the second or third base of the 3′ end. Four pairs of specific primers were developed to evaluate primer specificity and identify the optimal primers. These were used to assess the sensitivity of the mutation site and enable specific amplification of the *FecB* mutant gene. The designed primers for ARMS amplification are listed in Table 1. The PCR reaction mixture comprised 1 µL template DNA, 1 µL forward and reverse primers (10 μM), 0.2 µL DNA polymerase, 1 µL dNTP mixture (10 mM), 2.5 µL 10×Taq buffer (with MgCl_2_), and 18.3 µL ddH_2_O, for a total reaction volume of 25 µL. The expansion strips are as follows: 25 cycles of an initial 5 min denaturation at 95 °C, followed by 30 s at 94 °C, 30 s at 58 °C, and 30 s at 72 °C, with a final extension at 72 °C for 10 min. The specificity of the ARMS primers was evaluated using 1% agarose gel electrophoresis, enabling the identification of the best primers.

### 2.4. DNA Extraction and Validation of the Test Sample

Eighteen genomic ovary samples of small-tailed Han sheep were cryopreserved at −20 °C. The genomic DNA was extracted from the sheep ovarian tissue using a magnetic bead-based DNA extraction kit and stored at −20 °C. The best primer was used for ARMS amplification verification and the results were detected by 1% agarose gel electrophoresis. The PCR reaction system and expansion strips were the same as in Section 2.3.

### 2.5. Design and Preparation of Gold Nanomagnetic Bead (Fe_3_O_4_@Au)-Fluorescent Probe Complex

#### 2.5.1. Assembly of Gold Nanomagnetic Beads (Fe_3_O_4_@Au) with Linker-HS-SH

Linker freeze-dried powder containing disulfide bonds was centrifuged at 10,000 rpm for 2 min and then dissolved in sterilized duplex buffer to prepare a 100 µM Linker solution. The reducing agent was prepared by dissolving 4 mg of TCEP in 100 µL of 0.1 M Tris-HCl buffer (pH 7.5). The 100 µL Linker solution was thoroughly mixed with the reducing agent and left to react for 1 h. Next, 200 µL of 6% acetic acid and 50 µL of 3 M sodium acetate were added to the reduced Linker solution. Following this, 1.6 mL of anhydrous ethanol was added, and the mixture was centrifuged at 6000 rpm for 10 min using a high-speed centrifuge. After discarding the supernatant, 100 µL of Tris-HCl buffer (pH 7.5) was added and mixed thoroughly to prepare a 100 µM solution of Linker with successfully reduced sulfhydryl groups. An equal volume of Fe_3_O_4_@Au solution was transferred into a centrifuge tube and placed on a magnetic rack. The Fe_3_O_4_@Au particles were adsorbed at the bottom of the tube, and the supernatant was removed. The particles were rinsed three times by adding an equal volume of pH 7.5 Tris-HCl buffer and repeating the adsorption and removal steps. A 50 µM Linker solution was added to the centrifuge tube, mixed thoroughly, and incubated on an oscillator for 48 h. The tube was then placed on the magnetic rack again, and the solution was removed after the Fe_3_O_4_@Au particles were adsorbed at the bottom. The washing procedure was repeated thrice. Finally, the buffer was added to obtain the Fe_3_O_4_@Au-Linker solution.

#### 2.5.2. Y-Shaped Branches

The lyophilized powder of fluorescent reporter genes (Y-1, Y-2, and Y-3; see Table 1) was centrifuged at 10,000 rpm for 2 min, then dissolved in sterilized duplex buffer (pH 7.4) to a final concentration of 100 µM. Equal volumes of Y-1, Y-2, and Y-3 were mixed, heated to 95 °C for 3 min, and gradually cooled to room temperature to form Y. The prepared solution was stored at 4 °C for future use. The branches of L (L1 and L2) required no further processing.

#### 2.5.3. Fe_3_O_4_@Au-Linker-Cy5 Construction of Nanotree

**Figure 1:**
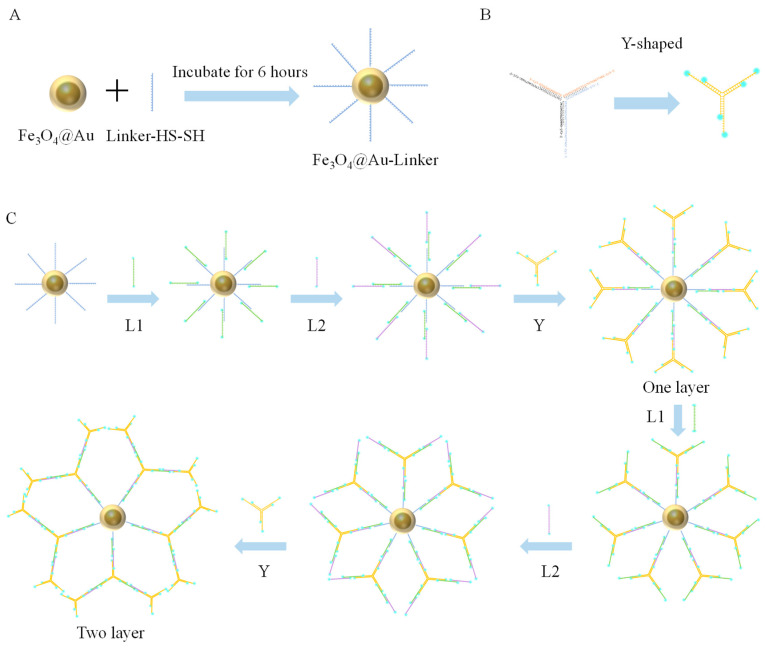
(**A**) Schematic diagram of assembly combination of Fe_3_O_4_@Au and Linker-HS-SH. (**B**) Y-shaped branch construction diagram. (**C**) Schematic diagram of construction process of Fe_3_O_4_@Au-Linker-Cy5 nanotree.

The 50 µM Fe_3_O_4_@Au-Linker solution, prepared as described in Section 2.5.1, was used for layer-by-layer self-assembly hybridization with complementary branches of L1, L2, and Y. Each hybridization step lasted 2 h. Multiple layers of Fe_3_O_4_@Au-Linker-Cy5-Nanotree were assembled. After each hybridization, unbound L1, L2, and Y were removed by magnetically adsorbing Fe_3_O_4_@Au onto the bottom of a centrifuge tube using a magnetic rack. The supernatant was discarded, and the pellet was washed thrice with an equal volume of 10 mM Tris-HCl (pH 7.4). Finally, the constructed Fe_3_O_4_@Au-Linker-Cy5-Nanotree was immersed in duplex buffer. The detailed procedure is shown in Figure 1.

### 2.6. Establishment of CRISPR-Cas12a Detection Method

The CRISPR-Cas12a assay was performed in a 20 µL reaction mixture containing 100 nM Cas12aRNP, 2 μL Fe_3_O_4_@Au-Linker-Cy5-Nanotree, 2 µL NE Buffer r2.1, 2 µL substrate DNA, and DNase/RNase-free water. The reaction proceeded at room temperature and was monitored using the Y040 multifunctional enzyme marker (Thermo Fisher Scientific, Waltham, MA, USA).

### 2.7. Accuracy Detection of CRISPR-ARMS Detection System

The genomic DNA was extracted from 18 small-tail Han sheep ovarian tissue using a magnetic bead-based DNA extraction kit and diluted to 200 ng/μL. Samples were numbered 1–18. The concentration and purity of substrate DNA were detected by agarose gel electrophoresis and spectrophotometer after ARMS amplification, and the CRISPR-Cas12a system was used to analyze all 18 samples, with wild-type (AA) and mutant homozygote (GG) controls employed to preliminarily determine genotypes. To verify the accuracy of CRISPR-ARMS detection, amplification products were analyzed by agarose gel electrophoresis, confirming the genotyping results.

## 3. Results

### 3.1. Fe_3_O_4_@Au-Linker-Cy5 Characterization of Nanotree

**Figure 2:**
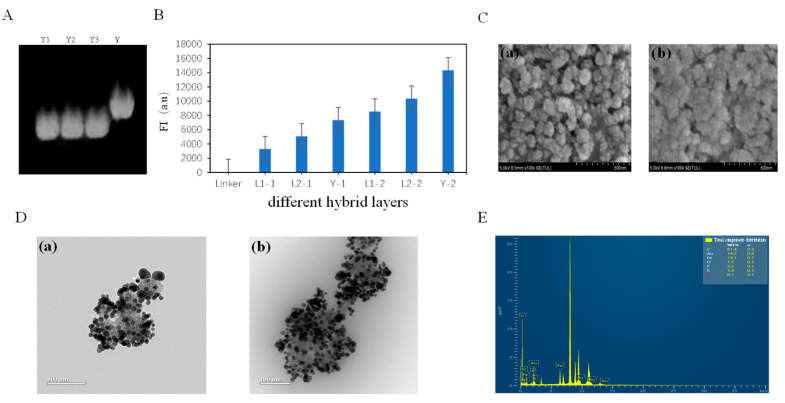
(**A**) Electropherograms of self-assembled Y-shaped branches. (**B**) The change of fluorescence intensity after Fe_3_O_4_@Au-Linker adding L1, L2, and Y. (**C**. **a**) SEM image of Fe_3_O_4_@Au, (**C**. **b**) SEM image of Fe_3_O_4_@Au-Linker-Cy5-Nanotree. (**D**. **a**) TEM image of Fe_3_O_4_@Au, (**D**. **b**) Fe_3_O_4_@Au-Linker-Cy5-Nanotree. (**E**) Total component distribution of Fe_3_O_4_@Au-Linker-Cy5-Nanotree including major elements such as C, N, P, O, Fe, and Au.

Attaching additional double- and multi-stranded fluorescence reporter genes to the surface of Fe_3_O_4_@Au-Linker enables the construction of Fe_3_O_4_@Au-Linker-Cy5 nanostructures, significantly enhancing the fluorescence sensor’s sensitivity and amplifying fluorescence signals. Due to the high cost of commercially synthesizing single-stranded DNA sequences longer than 150 nucleotides, DNA origami techniques were employed to develop complex DNA nanostructures. To this end, one Y-branch chain and two L-branch chains were designed and assembled onto Fe_3_O_4_@AuLinker in a layer-by-layer manner. The corresponding DNA sequences are listed in Table 1. Figure 2A presents the agarose gel electrophoresis results, confirming that the three complementary single-stranded DNA strands formed a stable Y-type DNA structure. Figure 2B shows the fluorescence intensity of Fe_3_O_4_@Au-Linker-Cy5-Nanotree after alternating the hybridization of Fe_3_O_4_@Au-Linker with complementary branches L1, L2, and Y over two layers. The fluorescence intensity of Fe_3_O_4_@Au-LinkerCy5-Nanotree increased progressively with the sequential addition of fluorescent reporter genes. Additionally, transmission electron microscopy (TEM) and scanning electron microscopy (SEM) were used to visualize the interactions between the gold nanomagnetic beads and fluorescent reporter genes. Following the nanotree assembly, magnetic beads were washed three times with PBS (pH 7.4, 10 mM) re-dispersed in 100 μL PBS, and a 2 μL drop of the solution was deposited on the carbon-coated side of the copper/carbon mesh. After air-drying at room temperature, the samples were characterized using TEM and SEM. Figure 2C,D depict SEM and TEM images of Fe_3_O_4_@Au before and after functionalization with fluorescence reporter nanocomposites. These images reveal the successful attachment of polymeric fluorescence reporter nanocomposites to the Fe_3_O_4_@Au surface. Figure 2E illustrates the whole-component distribution within the Fe_3_O_4_@Au-Linker-Cy5-nanotree, showing the presence of basic elements from reporter genes (C, P, and N) as well as the S element contributed by the linker after modification with fluorescence reporter nanocomposites.

### 3.2. CRISPR-ARMS System Optimization

#### 3.2.1. Optimization and Detection of ARMS Primers

**Figure 3:**
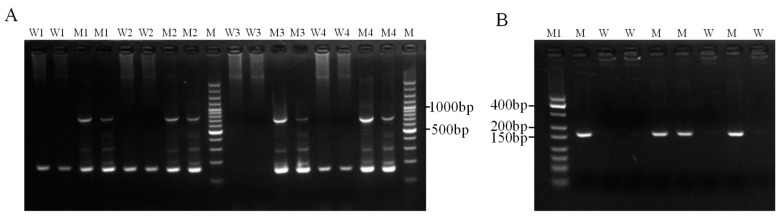
(**A**) Four ARMS primers (ARMS1, ARMS2, ARMS3, and ARMS4, respectively) were used to perform ARMS amplification on DNA samples of four gene sets (W1, W2, W3, and W4 represented wild-type samples amplified by ARMS1, ARMS2, ARMS3, and ARMS4 primers, respectively. M1, M2, M3, and M4 represented mutant samples amplified by ARMS1, ARMS2, ARMS3, and ARMS4 primers, respectively, and M represented marker). (**B**) ARMS amplification detection maps were performed on eight genomic DNA samples with primer ARMS3 (M represented mutant samples, W represented wild-type samples, and M1 represented marker).

To develop the CRISPR-ARMS method, we employed a stepwise optimization strategy. Since predicting the mutation site sensitivity of target genes in ARMS PCR is challenging, directly designing highly specific and efficient primers is difficult. To address this, we initially designed and synthesized four pairs of ARMS primers, with two pairs targeting wild-type and mutant sequences, respectively. Single-nucleotide mismatches were introduced at the penultimate and antepenultimate positions of each forward primer’s 3′ end, while all pairs shared the same reverse primer. The ARMS primer sequences are listed in Table 1. Sequenced wild-type (AA) and mutant (GG) genomic DNA from small-tailed Han sheep served as templates for ARMS PCR amplification using the four primer pairs, as described in Section 2.3. ARMS PCR products were analyzed using 1% agarose gel electrophoresis. The results showed that only the third primer pair (ARMS3) exhibited specificity, successfully amplifying target bands in mutant samples while showing no amplification in wild-type samples (Figure 3A). To further confirm the reliability and accuracy of ARMS3, we performed ARMS PCR amplification on eight additional samples (Figure 3B). The results demonstrated that ARMS3 specifically and consistently amplified mutant (GG/AG) samples with 100% accuracy and repeatability.

#### 3.2.2. Optimization of CRISPR-Cas12a System

**Figure 4:**
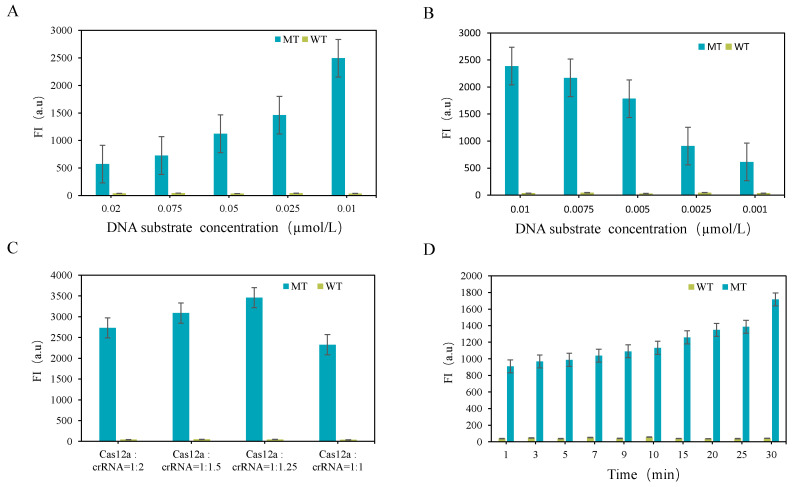
(**A**,**B**) Optimization of different substrate concentrations. (**C**) Molar ratio optimization of Cas12aRNP. (**D**) Cutting time of Cas12aRNP.

Optimizing fluorescence reporting systems is crucial for enabling CRISPR-ARMS-based detection. Improving the CRISPR-Cas12a reaction system can enhance detection sensitivity and significantly increase the distinction between wild-type and mutant types. Factors affecting the efficiency of the CRISPR-Cas12a system include DNA substrate concentration, Cas12a nuclease, crRNA, and Cas12a’s cutting time. To optimize these factors, we tested fluorescence intensity changes using DNA substrates at different concentrations (0.02 µM to 0.001 µM), Cas12aRNP with varying Cas12a:crRNA ratios (1:2, 1:1.5, 1:1.25, and 1:1), and cutting times of 0–30 min. For the DNA substrate concentration test, we fixed the Cas12aRNP concentration at 0.01 µM. The results in Figure 4A,B showed that both high and low DNA substrate concentrations decreased fluorescence intensity. The optimal DNA substrate concentration was 0.01 µM, which provided the highest detection sensitivity and cutting specificity. Therefore, 0.01 µM was selected as the final detection concentration. Figure 4C shows that the Cas12a to crRNA ratio of 1:1.25 produced the greatest fluorescence difference. Therefore, Cas12aRNP at 0.01 µM (Cas12a:crRNA = 1:1.25) was selected as the final cutting concentration. For the cutting time test, we aimed for rapid, high-sensitivity, and high-specificity detection of the *FecB* mutation gene. Figure 4D shows the fluorescence intensity over 1–30 min of reaction between Cas12aRNP and *FecB* with different genotypes. During this period, the fluorescence intensity did not increase significantly with longer reaction times. After 1 min, the fluorescence intensity of the mutant type was 26.6 times greater than that of the wild-type, indicating high detection sensitivity of the CRISPR-ARMS system. This suggests that detection time can be greatly shortened, enabling the rapid screening of *FecB* mutant genes.

### 3.3. Detection Results of CRISPR-ARMS System

**Figure 5:**
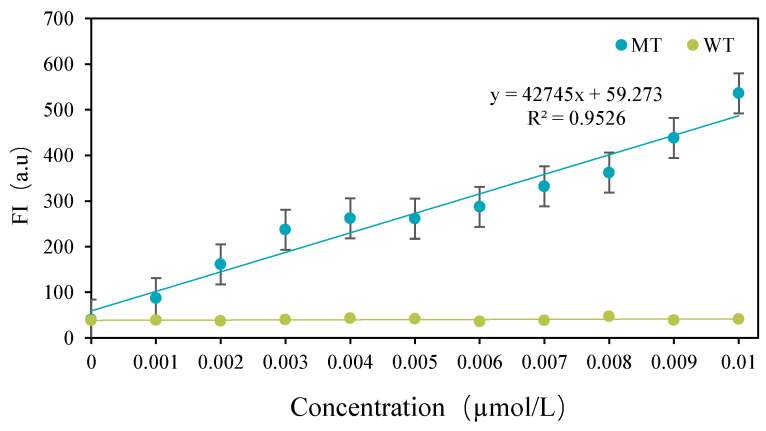
Linear regression curves for detection of different DNA substrate concentrations 0.001 µM L-0.01 µM.

Compared to the traditional ARMS amplification method, which requires agarose gel electrophoresis to detect specific amplification results, the ARMS + CRISPR-Cas12a system offers significant advantages. This method detects single-base mutations by completely inhibiting PCR amplification of wild-type sequences, without relying on the specificity of CRISPR-Cas12a. It achieves a lower detection limit and higher sensitivity than agarose gel electrophoresis, enabling the high-sensitivity screening of *FecB* mutated genes with simpler equipment and procedures. Figure 5 presents the linear regression curves for detecting varying concentrations of DNA substrates using the CRISPR-ARMS system. The linear regression equation is y = 42,754x + 59.273 (R^2^ = 0.9526), with a detection limit of 0.001 µM. The detection time was determined to be 1 min, and the results demonstrated a positive correlation between fluorescence intensity and DNA substrate concentration. At a DNA substrate concentration of 0.001µM, the fluorescence intensity decreased to 87 a.u. Below this concentration, the fluorescence values of the mutant reporting system approximated the background fluorescence of the wild-type fluorescence reporting system, making it difficult to distinguish between wild-type and mutant sequences. These findings confirm that the CRISPR-ARMS system can reliably distinguish DNA substrates at concentrations as low as 0.001 µM (0.02 pmol).

### 3.4. Actual Sample Testing and Verification

**Figure 6:**
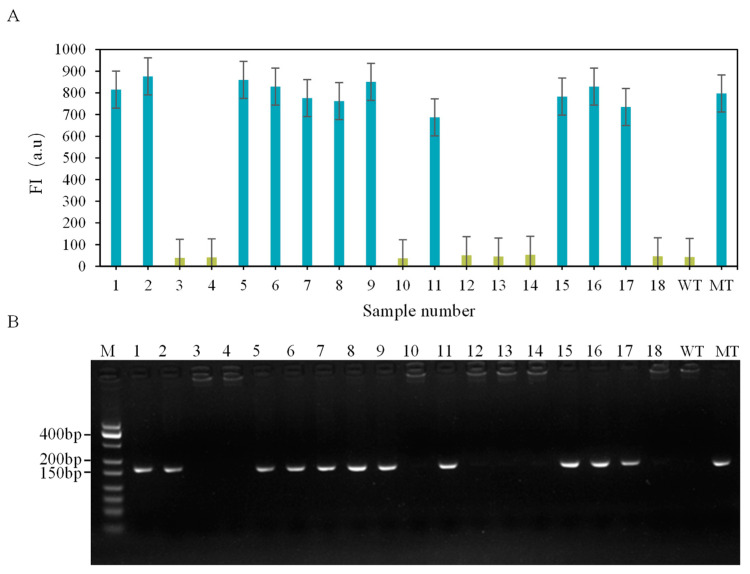
(**A**) CRISPR-ARMS to detect unknown genotype samples. (**B**) Electrophoretic map of unknown genotype samples.

The established CRISPR-ARMS system was applied to detect 18 samples with unknown genotypes, using known wild-type and mutant samples as controls. Genotypes were determined based on the fluorescence values of the reporter gene in the CRISPR-ARMS system. The results demonstrated that the system could accurately differentiate wild-type samples from mutant ones (Figure 6A). A comparison of the CRISPR-ARMS results with those from agarose gel electrophoresis (Figure 6B) confirmed 100% accuracy in detecting the *FecB* mutant gene in sheep.

## 4. Discussion

With advancements in genomics technology, detecting the *FecB* gene mutation in sheep has gained significant importance in modern animal husbandry. This study combines the CRISPR-Cas12a system with the ARMS amplification method, offering a novel and efficient approach for detecting complex DNA sequences. Cost is a critical factor in evaluating the practicality of any new technology. Compared to traditional PCR methods, the CRISPR-Cas12a system incurs higher reagent costs due to the Cas12a protein preparation and guide RNA synthesis [29]. Additionally, the specific primers required for the ARMS method add to the expenses. Although the initial investment is higher, the significant improvements in sensitivity and specificity provided by CRISPR-Cas12a could reduce economic losses from false negatives in the long term. Similarly, technologies like the modified DNA origami approach can enhance detection efficiency through signal amplification, potentially reducing testing time and further increasing cost-effectiveness. Therefore, detailed cost–benefit analyses tailored to various industries may be necessary to facilitate the widespread adoption of this technology.

The CRISPR-Cas12a system exhibits exceptional sensitivity in recognizing and cleaving target DNA, enabling the detection of mutations at very low copy numbers. When combined with the ARMS amplification method, it leverages a specific primer design to further enhance the specificity of mutation detection [30]. This synergy between sensitivity and specificity provides strong support for the early screening of *FecB* gene mutations. In terms of detection capacity, this system not only identifies known mutations but also has the potential to uncover novel mutation sites, demonstrating broader adaptability [31]. Compared to conventional PCR methods or SNP detection techniques, the combined CRISPR-Cas12a and ARMS approach significantly improves efficiency, sensitivity, and specificity. Traditional methods often involve longer processing times and labor-intensive procedures, whereas the new detection method delivers faster results [32]. This advancement optimizes the detection process and enables breeders to make timely decisions, thereby enhancing breeding management efficiency.

To expand the application potential of this technology, future research should focus on developing portable and integrated detection platforms to facilitate on-site testing [33]. Additionally, incorporating artificial intelligence [34] and big data analysis technologies [35] can further enhance the accuracy and efficiency of interpreting detection results.

## 5. Conclusions

To enhance the sensitivity of fluorescent biosensors and amplify the signal, a DNA nanotree structure combining gold nanomagnetic beads with fluorescent reporter genes was designed using “similar DNA origami technology”. The CRISPR-ARMS system relies on specific amplification rather than the precise cleavage activity of CRISPR-Cas12a. It can detect concentrations as low as 0.001 µM within one minute, without requiring complex sample preparation, specialized equipment, labor-intensive procedures, or advanced analytical techniques. This method offers the advantages of speed, low labor intensity, high accuracy, and high sensitivity. By simply tuning the ARMS amplification primer and crRNA, the system shows promise as an innovative platform for detecting a wide range of single-base mutations. Concurrently, the CRISPR-ARMS system can be integrated with technologies such as colorimetry and electrochemistry, providing technical support for both laboratory and field testing. With further development, this CRISPR-Cas12a-based platform holds significant potential for diverse applications.

## Figures and Tables

**Table 1:** Primers, crRNA, and ssDNA probe sequences used in the experiment.

Primer Name	Sequence (5′ → 3′)
PCR1-F	AACATTAAACAATCTGTAGTGCCGT
PCR1-R	CATAGCGGATCTAGACAGATGAGTT
ARMS1-F	GGTTCCGAGAGACAGAAATATATGA
ARMS1-R	TATTGTATTTGCATTTCCTGTCCCT
ARMS2-F	GGTTCCGAGAGACAGAAATATAACA
ARMS2-R	TATTGTATTTGCATTTCCTGTCCCT
ARMS3-F	GGTTCCGAGAGACAGAAATATATGG
ARMS3-R	TATTGTATTTGCATTTCCTGTCCCT
ARMS4-F	GGTTCCGAGAGACAGAAATATAACG
ARMS4-R	TATTGTATTTGCATTTCCTGTCCCT
Linker-HS-SH	HS-SH-CGATTGACTCTCTTTTTTTT
L1	Cy5-GAGAGTCAATCGTTTTTTTTTCTATTCGCAGTAGAT-Cy5
L2	Cy5-GAGAGTCAATCGTTTTTTTTATCTACTGCGAATAGA-Cy5
Y1	Cy5-CGATTGACTCTCCACGCTGTCCTAACCATGACCGTCGAAG-Cy5
Y2	Cy5-CGATTGACTCTCCTTCGACGGTCATGTACTAGATCAGAGG-Cy5
Y3	Cy5-CGATTGACTCTCCCTCTGATCTAGTAGTTAGGACAGCGTG-Cy5
crRNA	ATGCCTCATCAACACCGTCC

## Data Availability

Data are contained within the article.
